# Unusual Presentation of Atrial Flutter With Slow Ventricular Response

**DOI:** 10.7759/cureus.15801

**Published:** 2021-06-21

**Authors:** Varun Dobariya, Ebubechukwu Ezeh, Mohamed S Suliman, Davinder Singh, Samson Teka

**Affiliations:** 1 Internal Medicine, Marshall University, Joan C. Edwards School of Medicine, Huntington, USA

**Keywords:** bradycardia, atrial flutter, intrinsic av nodal disease, tachycardia, slow ventricular response

## Abstract

Atrial flutter is usually associated with tachycardia with a ventricular rate of 150 beats per minute. Less commonly, it may be associated with a slow ventricular response (SVR). This is typically seen in patients taking atrioventricular (AV) nodal blocking agents such as beta-blockers. In the absence of these drugs, atrial flutter with SVR may suggest intrinsic AV nodal disease, electrolyte disturbances, or atrial disease. We present a case of atrial flutter with SVR in a patient who was not receiving AV nodal blocking agents.

## Introduction

Although less common, atrial flutter occurs in many of the same situations as atrial fibrillation. It may represent a stable rhythm or a bridge arrhythmia between sinus rhythm and atrial fibrillation [1]. It may be symptomatic or asymptomatic. When symptomatic, common presentations include palpitations, shortness of breath, fatigue, lightheadedness, as well as an increased risk of atrial thrombus formation that may cause cerebral and/or systemic embolization [1].

Electrocardiography (ECG) remains the mainstay of diagnosis. It shows the characteristic negative sawtooth flutter waves in the inferior leads. Atrial flutter typically causes tachycardia. Less commonly, it may also be associated with a normal heart rate (HR) or even bradycardia, as seen in our patient [2]. Thus, clinicians need to always be aware of this and remember that bradycardia does not rule out a diagnosis of atrial flutter. This paper was previously presented as a poster during the American College of Physicians West Virginia chapter meeting in 2020.

## Case presentation

**Figure 1:**
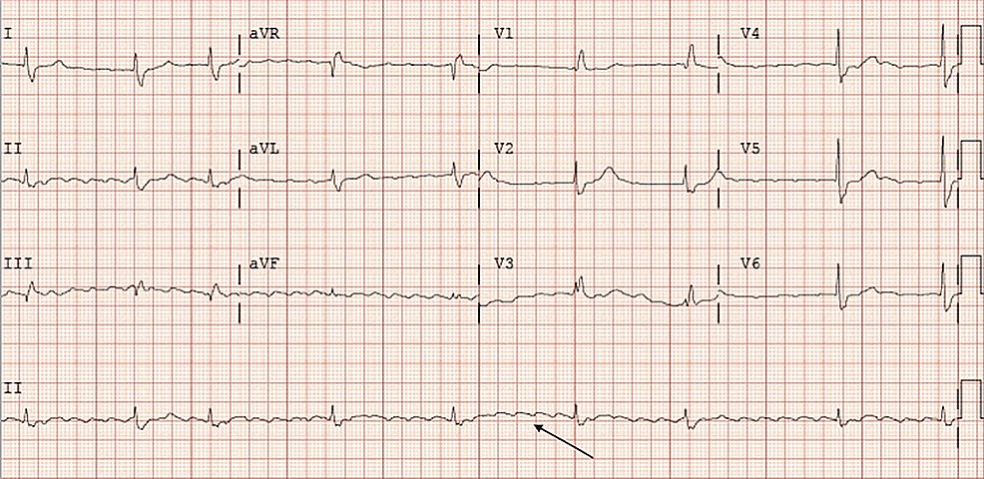
Atrial flutter (arrow).

A 77-year-old male with a history of coronary artery disease status post-percutaneous coronary intervention with two stents in-situ, hypertension, and type 2 diabetes mellitus was transferred from an outside facility on account of a one-week history of HRs ranging between 30 and 40 beats per minute. Home medications included amlodipine, aspirin, atorvastatin, clopidogrel, metoprolol, and sublingual nitroglycerin on an as-needed basis. The patient stated that he had not taken his metoprolol for five days prior to the presentation. He also reported chronic dyspnea, unchanged from his baseline. He denied any chest pain, palpitations, or dizziness. On presentation, his vitals were unremarkable with a blood pressure of 146/56 mmHg and HR of 44 beats per minute. Initial labs and imaging workup were unremarkable except for a serum magnesium level of 1.7 mg/dL. On arrival, ECG revealed atrial flutter with variable block ranging from 2:1 to 6:1, with a ventricular rate of 47 beats per minute and a right bundle branch block (RBBB). The initial ECG is shown in Figure 1.

**Figure 2:**
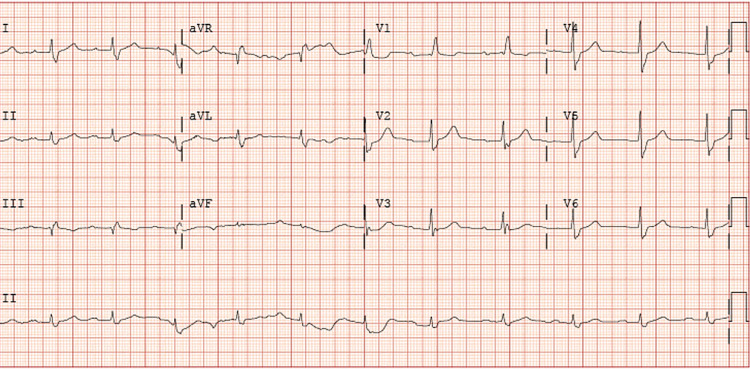
Sinus rhythm with first-degree atrioventricular block.

Magnesium was replaced but the patient’s ECG remained unchanged. His echocardiogram was unremarkable. Anticoagulation was started with apixaban. Electrophysiology (EP) was consulted. Intracardiac echocardiogram-guided radiofrequency ablation (RFA) of the cavotricuspid isthmus (CTI) was performed. Subsequent post-procedure ECG showed sinus rhythm with first-degree block and RBBB with ventricular rate improving to 60-70 beats per minute. The post-ablation ECG is shown in Figure 2. The patient’s HR remained within the normal range post-procedure. He was subsequently discharged home on his home medications except for metoprolol which was held. He was given an appointment to follow up with EP as an outpatient.

## Discussion

In patients who are not taking atrioventricular (AV) nodal blockers, the atrial rate in atrial flutter typically ranges from 240 to 300 beats per minute with a regular ventricular rate of approximately 150 beats per minute [3]. However, the ventricular rate may be slower in the presence of atrial disease or antiarrhythmic drugs [2]. Thus, the clinical presentation of atrial flutter largely depends on the ventricular rate [4].

The action potential of the AV node depends on calcium ions flowing through the kinetically slow channel. It has been called a “slow response” tissue. Even input/output ratios in atrial flutter (e.g., 2:1 or 4:1 conduction) are more common than odd ratios (e.g., 3:1 or 5:1). Odd ratios are thought to reflect a bilevel block in the AV node. Sometimes, variable conduction may occur with alternating or seemingly random patterns of 2:1, 3:1, 4:1, or other conduction patterns due to varying levels of block in the AV node [3]. This was the case in our patient who had conduction pattern ranging from 2:1 to 6:1.

Atrial flutter with AV node blockade is a potentially life-threatening cause of bradycardia usually seen in patients with preexisting valvular or structural diseases and/or conduction system disease [5]. Patients may also present with hemodynamic instability from the slow ventricular response (SVR) [6]. As stated earlier, a higher degree of AV block with resulting lower ventricular conduction may indicate the use of AV nodal blocking drugs, intrinsic conducting system disease, electrolyte abnormalities, or hypothermia. Our patient was off metoprolol for almost a week prior to presentation making it an unlikely cause of his SVR [6]. In addition, hypomagnesemia was ruled out as a possible cause when replacement failed to correct the SVR.

ECG is the mainstay of diagnosis. Our patient’s ECG showed the characteristic pattern of sawtooth negative flutter waves in the inferior leads [3]. An echocardiogram is also indicated in new-onset atrial flutter to rule out structural heart disease. An echocardiogram may also diagnose the rare cardiac lymphoma which has been reported as a cause of atrial flutter with SVR [7]. Treatment modalities include pharmacologic or RFA of the CTI. One study comparing both treatment modalities found that RFA of the CTI was associated with greater restoration of sinus rhythm, as was the case in our patient. RFA of the CTI is also associated with fewer hospitalizations, greater sense of well-being, and a lower incidence of atrial fibrillation compared to pharmacologic measures [8]. Therefore, RFA of the CTI should be performed when feasible.

## Conclusions

Even though the ventricular rate in atrial flutter is usually 150 beats per minute, the rate may be slower in the presence of atrial disease, AV nodal disease, electrolyte imbalances, or antiarrhythmic drugs. Uncommonly, there can be slow AV nodal conduction with a variable or higher degree of block in the absence of drugs that reduce AV nodal conduction. This may suggest co-existing AV nodal disease, as was the case in our patient. Clinicians should always remember this presentation when assessing patients with atrial flutter who do not have tachycardia. RFA of the CTI may be curative.
